# Adherence to driving cessation advice given to patients with cognitive impairment and consequences for mobility

**DOI:** 10.1186/s12877-018-0910-4

**Published:** 2018-09-17

**Authors:** Dafne Piersma, Anselm B. M. Fuermaier, Dick De Waard, Ragnhild J. Davidse, Jolieke De Groot, Michelle J. A. Doumen, Rudolf W. H. M. Ponds, Peter P. De Deyn, Wiebo H. Brouwer, Oliver Tucha

**Affiliations:** 1 Department of Clinical and Developmental Neuropsychology, University of Groningen, Groningen, The Netherlands; 2 SWOV Institute for Road Safety Research, The Hague, The Netherlands; 3 Department of Psychiatry and Neuropsychology, School of Mental Health and Neurosciences (MHeNS), Maastricht University, Maastricht, The Netherlands; 4 Department of Neurology and Alzheimer Research Center, University of Groningen and University Medical Center Groningen, Groningen, The Netherlands

**Keywords:** Dementia, Driving cessation, Adherence to driving cessation advice, Alternative transportation, Mobility

## Abstract

**Background:**

Driving is related to social participation; therefore older drivers may be reluctant to cease driving. Continuation of driving has also been reported in a large proportion of patients with cognitive impairment. The aim of this study is to investigate whether patients with cognitive impairment adhere to driving cessation advice after a fitness-to-drive assessment and what the consequences are with regard to mobility.

**Methods:**

Patients with cognitive impairment (*n* = 172) participated in a fitness-to-drive assessment study, including an on-road driving assessment. Afterwards, patients were advised to either continue driving, to follow driving lessons, or to cease driving. Approximately seven months thereafter, patients were asked in a follow-up interview about their adherence to the driving recommendation. Factors influencing driving cessation were identified using a binary logistic regression analysis. Use of alternative transportation was also evaluated.

**Results:**

Respectively 92 and 79% of the patients adhered to the recommendation to continue or cease driving. Female gender, a higher Clinical Dementia Rating-score, perceived health decline, and driving cessation advice facilitated driving cessation. Patients who ceased driving made use of less alternative modes of transportation than patients who still drove. Nonetheless, around 40% of the patients who ceased driving increased their frequency of cycling and/or public transport use.

**Conclusions:**

Adherence to the recommendations given after the fitness-to-drive assessments was high. Female patients were in general more likely to cease driving. However, a minority of patients did not adhere to driving cessation advice. These drivers with dementia should be made aware of the progression of their cognitive impairment and general health decline to facilitate driving cessation. There are large differences in mobility between patients with cognitive impairment. Physicians should discuss options for alternative transportation in order to promote sustained safe mobility of patients with cognitive impairment.

## Background

Continuation of driving after being diagnosed with dementia has been found repeatedly [1–10]. Nevertheless, with the progression of the disease, cognitive abilities needed for safe driving gradually decrease and driving cessation is likely to become inevitable [11, 12]. It is difficult to define when a patient with dementia is no longer fit to drive [13] because of large individual differences in the patterns of dysfunctions, related to the different aetiologies of dementia [14, 15]. Therefore, the most appropriate moment to cease driving needs to be assessed on a case-by-case basis [16].

The decision to cease driving is not easily made as driving is associated with social participation, independence, and well-being [17, 18]. Some patients with dementia cease driving suddenly, e.g. from one day to another, or as a result of an accident, diagnosis, or other critical event, while others cease driving gradually [19]. These patients may drive less kilometres (i.e. driving reduction) or avoid difficult driving situations (i.e. driving restriction) before ceasing driving entirely [19]. However, a proportion of patients with dementia continues to drive despite evidence of a decreased fitness to drive [20]. Some of these patients did not recall their fitness-to-drive assessment, others were not aware of their own cognitive impairment (due to decreased insight associated with dementia) or believed that their cognitive impairment did not affect driving safety [13, 16, 21–25]. According to the last group, the assessment process was ‘not fair’ and did not accurately reflect their fitness to drive [13, 16, 21]. These findings suggest that fitness-to-drive assessments should be comprehensive, comprising several types of tasks and sources of information, and that guidance for patients with dementia in interpreting a recommendation about driving is essential [25–28].

The process of driving cessation is affected by intrapersonal, interpersonal, and environmental factors [29]. Intrapersonal factors are factors related to the driver, interpersonal factors are derived from relationships with others involved in decisions about driving, and environmental factors are external influences not associated to the driver or the relationship with others.

Intrapersonal factors include, among others, age, gender, the presence and awareness of decline in physical, visual, and cognitive abilities as well as an opinion regarding the importance of driving and one’s own driving safety. With increasing age, driving cessation becomes more likely [30], especially females are more likely to cease driving than men, even prematurely [31, 32]. An important reason for driving cessation among older drivers is perceived health decline, in particular in vision and cognition [10, 22, 30, 31, 33–38]. Cognitive impairment is strongly associated with various aetiologies of dementia that are characterized by distinct symptoms and impairments, therefore driving cessation might be more likely in one or the other aetiology of dementia. Seiler and colleagues [9] reported that as many as 90.9% of the patients with dementia with Lewy bodies (DLB) ceased driving whereas only about 55–65% of the patients with Alzheimer’s disease (AD), vascular dementia (VaD) and frontotemporal dementia (FTD) ceased driving. Furthermore, older people reported other reasons for driving cessation such as no need to drive anymore (e.g. because of retirement), decreased confidence while driving or lack of enjoyment during driving, and costs of fuel and upkeep of the car [18, 34, 39–41].

Interpersonal factors comprise the opinions of family members and authority figures about the patient’s driving safety. Family members may encourage driving cessation by expressing concerns about driving safety or even by taking away the keys [9, 18], however, about half of the family members with doubts about the patient’s driving safety were found not to attempt promoting driving cessation [42]. If family members do bring up the topic, older drivers may not be willing to follow up their advice [18]. Moreover, there is a minority of family members who encourage continuation of driving because they believe the patient still drives safely or they benefit from the patient’s driving [11, 22, 23, 41]. In the majority of cases, patients with dementia and their family members need support from physicians regarding counselling and evaluation of the patient’s fitness to drive [13, 18]. There are indications that recommendations to cease driving from authority figures, such as physicians, facilitate driving cessation [18, 22, 39, 42].

Environmental factors include traffic accidents and availability of alternative transportation. Traffic accidents and near misses have been reported as reasons for driving cessation [9, 22, 29]. Nevertheless, some patients with dementia continue driving for up to three years after experiencing a traffic accident [40, 43]. Additionally, *not* having caused any accident may also be a reason to continue driving [29]. Byszewski and colleagues [27] suggested that discussing alternative transportation may enhance acceptance of driving cessation, but mixed results have been obtained about the use of alternative transportation by patients with cognitive impairment. Talbot and colleagues [30] reported that patients living in a city, i.e. where alternative modes of transport are available, are more likely to cease driving. However, Taylor and Tripodes [44] found that the majority of patients with dementia may depend on rides of their partners, relatives, or friends and observed no increase in walking, using public transport, taxis, or van services after driving cessation.

This study has four aims. The first aim of this study is to evaluate how many patients with dementia adhere to the recommendation given after a fitness-to-drive assessment. The second aim is to identify which factors play a role in driving cessation of patients with dementia who underwent a fitness-to-drive assessment. Based on the literature, major factors hypothesized to be related to driving cessation are increasing severity of cognitive impairment and recommendations to cease driving. The third aim is to investigate whether patients with different aetiologies of dementia show a different likelihood of driving cessation. Based on the study of Seiler and colleagues [9], patients with DLB are expected to cease driving more frequently compared to patients with other aetiologies of dementia. The final aim is to evaluate transportation options for patients with dementia beyond driving. Eventually, implications will be provided of how driving cessation and alternative transportation could be addressed in clinical practice.

## Methods

### Participants

Participants with cognitive impairment were recruited via multiple health care centres and from the general community. Inclusion criteria were an age above 30, a diagnosis of mild cognitive impairment, dementia, or Parkinson’s disease (PD) with self-reported cognitive decline, a current valid driver’s licence and a wish to continue driving. Exclusion criteria were the diagnosis of other neurological or psychiatric conditions that may influence driving performance and usage of medications with a severe influence on driving ability (International Council on Alcohol, Drugs and Traffic Safety Category III). Since not all participants had a diagnosis of dementia, they will be referred to as patients with cognitive impairment.

One hundred and seventy-two patients with cognitive impairment completed the study. Patients were aged 49 to 91 years (mean = 71.3 years; SD = 8.8 years) and 128 (74.4%) of the patients were men. Patients had held a driver’s licence for 11 to 73 years (mean = 49.7 years; SD = 9.0 years) and the estimation of their total distance driven ranges from 87,000 to 12,183,000 km (mean = 1,720,000 km; SD = 2,692,000 km). Eighty-three (48.3%) patients were diagnosed with AD, 15 (8.7%) with VaD, 10 (5.8%) with AD and VaD, 13 (7.6%) with FTD, 8 (4.7%) with DLB, 17 (9.9%) with PD and 12 (7.0%) with other aetiologies of cognitive impairment. The aetiology of cognitive impairment was unclear in 14 (8.2%) cases.

### Measures

The measures used for the present study represent a selection of measures as obtained from a comprehensive fitness-to-drive assessment following the protocol as described by Piersma and colleagues [1]. The preselection of measures was based on the literature and intended to cover relevant factors for driving cessation [10, 11, 13, 18, 22, 27, 29–35, 37, 39, 41, 42, 44].

#### Intrapersonal factors

Intrapersonal factors used for the prediction of driving cessation included age, gender, diagnosis (AD vs. other), level of cognitive impairment, decline in health, visual acuity (range 0–1), visual contrast sensitivity (range 0–16), importance of driving for the individual patient, and the opinion of patients about their own driving safety. The level of cognitive impairment was measured by the total score of the Clinical Dementia Rating (CDR) scale [45] and the total score of the Mini-Mental State Examination (MMSE) [46, 47]. Decline in health was determined by asking the patients during a follow-up interview whether they experienced changes in their health since their fitness-to-drive assessment. Answers were coded into three categories: (1) no, (2) to some extent, and (3) yes. During clinical interviews, patients were asked whether driving was important to them. Answer options were: (1) very important, (2) important, (3) practical but not important, and (4) unimportant. During the same interviews, patients were asked how they experienced their driving safety. Answers were divided into three categories: (1) still driving as safely as when they were middle-aged, (2) driving less safely compared to when they were middle-aged or (3) driving unsafely.

#### Interpersonal factors

Interpersonal factors included the recommendation given by a researcher after the fitness-to-drive assessment, whether an authority figure (e.g. physician, driving instructor) recommended driving cessation, and the opinion of an informant about the patient’s driving safety. The recommendation after completion of the fitness-to-drive assessment was given by one of the researchers involved and represented either (1) cease driving, (2) follow driving lessons and sign up for an official relicensing procedure or (3) continue driving. Besides the recommendation of a researcher after the fitness-to-drive assessment, also a recommendation to cease driving from an authority figure could be reported during the follow-up interview. Lastly, the opinion of an informant about the driving safety of the patient was asked during a clinical interview. Answers were divided into three categories: (1) still driving as safely as when the patient was middle-aged, (2) driving less safely compared to when the patient was middle-aged or (3) driving unsafely.

#### Environmental factors

Three environmental factors were considered, i.e. the opportunity to be passenger of another private car (*yes* or *no*), the number of other modes of transport used (e.g. walking, cycling, public transport, and taxis), and the number of car accidents. Accidents included accidents in the twelve months prior to study participation and (almost) accidents after the fitness-to-drive assessment prior to the follow-up interview.

#### Indications of driving reduction, restriction, and cessation

Driving reduction and restriction were considered as indications of a process of driving cessation. The variables were based on questions in a driving questionnaire. Driving reduction was derived from the patients’ estimations of their driving experience in the previous twelve months minus the patient’s estimations of their average driving experience per year since they obtained their driving licence. The questions for driving experience had the following answer options: (1) less than 1.000 km, (2) 1.000–5.000 km, (3) 5.000–10.000 km, (4) 10.000–20.000 km, (5) 20.000–30.000 km, (6) 30.000–50.000 km, (7) more than 50.000 km. Driving restriction was calculated by summing up the number of driving situations that were being avoided (range 0–9). The patients answered a multiple-choice question: ‘Do you attempt to avoid the following traffic situations?’. Answer options were *peak hours/crowded roads, motorways, adverse weather conditions (like rain, fog or snow), slippery roads/snow on the road, driving when it is dark, turning left, driving unfamiliar roads, driving abroad, another traffic situation,* and *none*. The final outcome measure was whether the patient was still driving or not (*StillDriving*), which was asked during a follow-up interview.

### Procedure

Patients with cognitive impairment participated on a voluntary basis. Patients received no direct reward for participation, but patients who passed the on-road driving assessment could use this outcome in an official relicensing procedure. Failing the on-road driving assessment did *not* lead to revocation of the patients’ driving licences.

The fitness-to-drive assessment consisted of two sessions. On the first occasion, clinical interviews with the participant and an informant were conducted, as well as a comprehensive neuropsychological assessment and driving simulator rides. Participants invited an informant of their choice, usually their partner. During the first session, participants were also screened to assure that they met the minimum legal requirements for an on-road driving assessment with regard to visual functions (visual acuity of 0.5, horizontal field of view of 120 degrees) and motor functions (no major impairments of both hands, or legs). The first session lasted approximately four hours in total, including around half an hour driving simulation. On the second occasion, the on-road driving assessment took place, which lasted around 45 min.

After the fitness-to-drive assessment, a driving recommendation was given by one of the researchers involved based on both the off-road and on-road assessments as well as clinical judgment. If patients were recommended to continue driving, this was communicated via postal mail. These patients received an overview of their personal fitness-to-drive assessment results corroborated with an explanation of the findings and the recommendation in writing. If patients were recommended to follow driving lessons or to cease driving, they were called and invited for an appointment with a neuropsychologist to discuss the results and the recommendation. After this appointment, these patients also received an overview of their personal fitness-to-drive assessment results, an explanation of the findings, the recommendation, and a summary of the conversation with the neuropsychologist in writing.

The follow-up interview took place by telephone three to twenty months (M = 7.3 months, SD = 3.6 months) after participation in the fitness-to-drive assessment. Questions were asked to the patient (*n* = 78), to the patient and the patient’s partner together (*n* = 29) or to an informant only (*n* = 65). Informants were the partners of the patients (*n* = 57), or other relatives. Questions regarded whether the health of the patient declined, whether or not the patient ceased driving including reasons for this choice as well as use of alternative transportation. This interview lasted around 30 min per patient.

### Statistical analyses

Values were missing in less than 3% of cases per variable, and were not replaced.

#### Adherence to the recommendation

Adherence to the recommendations given after the fitness-to-drive assessment was investigated using driving cessation rates and information from the follow-up interview on whether patients followed driving lessons and signed up for an official relicensing procedure. Reported reasons for non-adherence were recorded.

#### Factors related to driving cessation

Factors related to driving cessation were explored in two ways, i.e. first by describing reported reasons for driving cessation in the follow-up interviews using percentages and second by predicting driving cessation in a logistic regression analysis. Current and retired drivers were statistically compared on predictor variables. These variables included intrapersonal factors, interpersonal factors, environmental factors, and two factors related to the process of driving cessation (see Measures). Predictor variables correlating significantly (*p* < 0.05) (point biserial correlation coefficients) with *StillDriving* were selected for the binary logistic regression analysis with forced entry of predictor variables.

#### Driving cessation per aetiology

To evaluate differences in driving cessation rates between patients with different aetiologies of cognitive impairment, the numbers and percentages of patients who ceased driving at follow-up were calculated per aetiology.

#### Mobility of patients with cognitive impairment

It was examined which modes of transport were important for patients with cognitive impairment to continue to use and which modes of transport were used by current and retired drivers. In addition, changes in frequencies of walking, cycling, and public transport use after the fitness-to-drive assessment were compared between current and retired drivers based on the question “Do you walk/cycle/use public transport less or more since the fitness-to-drive assessment?”. Finally, reasons for *not* walking, cycling, or using public transport were examined.

## Results

### Adherence to the recommendation

The vast majority of patients who were recommended to continue driving adhered to this recommendation (92.4%) (Table 1). Six (7.6%) patients decided to cease driving for one or two reasons: family members advocated driving cessation (*n* = 3), the patient felt driving was no longer safe (*n* = 2), an authority figure recommended driving cessation (*n* = 1), perceived health decline (*n* = 1), perceived stress related to the official relicensing procedure (*n* = 1), feeling uncomfortable driving or afraid to drive (*n* = 1), and a near miss occurred (*n* = 1).

**Table 1:** Driving continuation and cessation by patients with cognitive impairment per recommendation given after the fitness-to-drive assessment

Recommendation	Driving at follow-up	
	Yes	No
Continue driving (*n* = 79)	73 (92.4%)	6 (7.6%)
Driving lessons (*n* = 31)	18 (58.1%)	13 (41.9%)
Cease driving (*n* = 62)	13 (21.0%)	49 (79.0%)
Total (*n* = 172)	104 (60.5%)	68 (39.5%)

Thirty-one patients with cognitive impairment were recommended to follow driving lessons and sign up for the official relicensing procedure. Of the thirteen patients who ceased driving, one (7.7%) patient followed driving lessons, but was recommended to cease driving by the driving instructor, and two (15.4%) patients signed up for the official relicensing procedure. The procedure was still pending for one patient while the other patient failed the on-road driving assessment for driving license renewal. Of the eighteen patients who were still driving, twelve (66.7%) patients followed driving lessons and eight (44.4%) patients signed up for the official relicensing procedure. This procedure was still pending in five cases, and three patients renewed their driving license. Five patients who continued to drive (27.8%) did not follow driving lessons and also did not sign up for the official relicensing procedure. Notably, several patients reported that they restricted or reduced their driving after the fitness-to-drive assessment. Moreover, two patients had planned to sign up for the official relicensing procedure in a few months depending on their health status.

The majority of patients with cognitive impairment who were recommended to cease driving, adhered to this recommendation (79.0%). Nevertheless, thirteen patients did not. Two of them were considering driving cessation and reduced driving very much already. One more patient was willing to cease driving in the future, when the partner would advocate driving cessation. However, ten patients were not considering to cease driving at all, with five patients giving reasons for driving continuation (driving is going well (*n* = 2), having a partner as co-pilot (*n* = 2), because of mobility needs (*n* = 1)).

### Factors related to driving cessation

#### Reported reasons for driving cessation

**Fig. 1:**
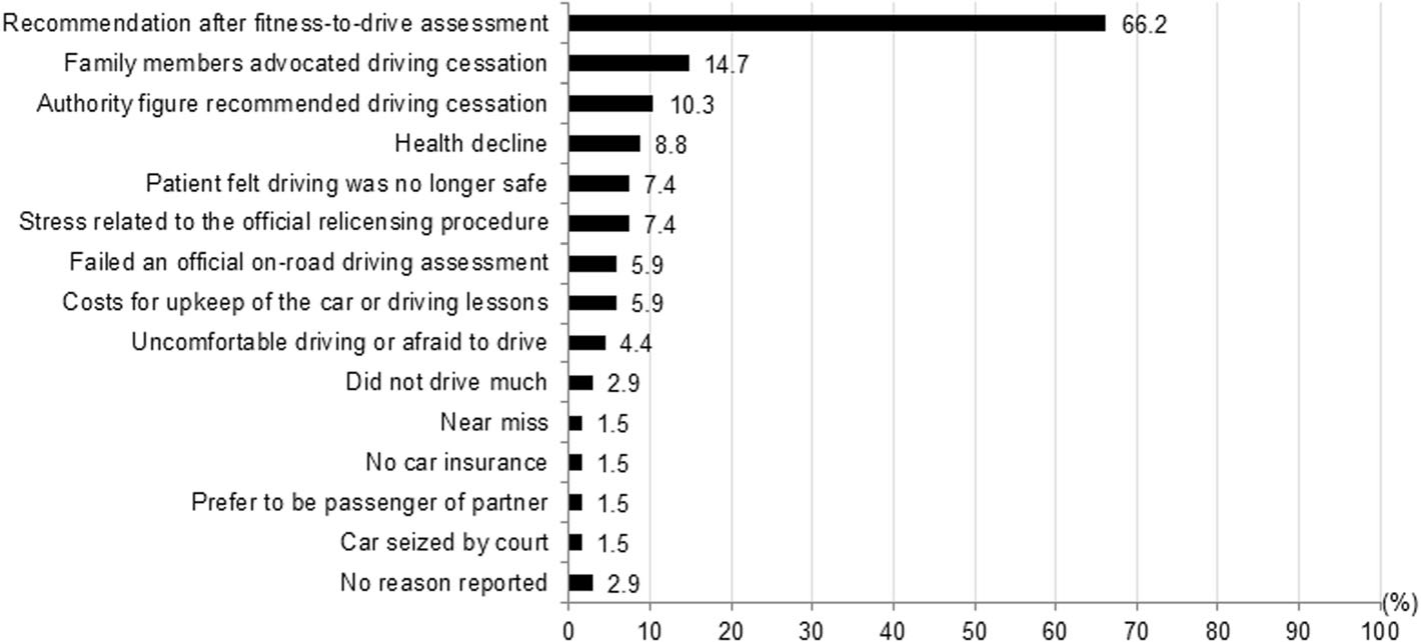
Percentages of reported reasons for driving cessation by patients with cognitive impairment who ceased driving (multiple answers possible, *n* = 68)

Patients with cognitive impairment reported one up to five reasons for driving cessation (Fig. 1). Two patients who were not driving did not report a reason for driving cessation, since they did not make a definite choice about whether they would never drive anymore.

#### Prediction of driving cessation

Retired drivers were significantly older, had more often a diagnosis of AD, a higher CDR-score, a lower MMSE-score, more pronounced health decline, and a lower visual contrast sensitivity than current drivers (Table 2). Moreover, retired drivers were more often recommended to cease driving, both after the fitness-to-drive assessment and by authority figures, than current drivers. Furthermore, retired drivers used less alternative modes of transport than current drivers. Lastly, trends (.05 < *p* < .10) were found for retired drivers being more often female, finding driving less important, and being more often a passenger of other car drivers than current drivers.

**Table 2:** Comparison of current and retired drivers with cognitive impairment on predictor variables

	Group		*p* Value (df)
	Current drivers (*n* = 104)	Retired drivers (*n* = 68)	
Intrapersonal factors			
Age in years, mean (SD), y	70.2 (8.7)	73.0 (8.7)	.032 (171)^a^*
Male sex, No. (%)	83 (79.8%)	45 (66.2%)	.051 (1)^b^
Diagnosis of AD, No. (%)	53 (51.0%)	40 (58.8%)	.035 (1)^b^*
CDR-score, No. (%)			
0	15 (14.4%)	1 (1.5%)	<.001 (2)^c^*
0.5	86 (82.7%)	44 (64.7%)	
1	3 (2.9%)	23 (33.8%)	
MMSE-score, mean (SD)	24.9 (3.5)	22.4 (4.2)	<.001 (171)^a^*
Health decline, No. (%)			
No	76 (73.1%)	33 (49.2%)	.004 (2)^c^*
To some extent	7 (6.7%)	5 (7.5%)	
Yes	21 (20.2%)	29 (43.3%)	
Visual acuity (0–1), mean (SD)	.88 (0.21)	.84 (0.21)	.181 (169)^a^
Contrast sensitivity (0–16), mean (SD)	12.84 (0.68)	12.55 (0.96)	.022 (170)^a^*
Importance of driving, mean (SD)	1.57 (0.73)	1.78 (0.83)	.091 (171)^a^
Patient’s judgement of driving safety, No. (%)			
Safe	88 (85.4%)	52 (76.5%)	.136 (2)^c^
Less safe than when middle-aged	15 (14.6%)	16 (23.5%)	
Unsafe	0 (0.0%)	0 (0.0%)	
Interpersonal factors			
Recommendation given after fitness-to-drive assessment, No. (%)			
Continue driving	73 (92.4%)	6 (7.6%)	<.001 (2)^c^*
Driving lessons	18 (58.9%)	13 (41.9%)	
Cease driving	13 (21.0%)	49 (79.0%)	
Authority figure recommended driving cessation, No. (%)	1 (1.0%)	12 (17.6%)	<.001 (1)^b^*
Informant’s judgement of driving safety, No (%)			
Safe	68 (66.6%)	42 (64.6%)	.190 (2)^c^
Less safe than when middle-aged	32 (31.4%)	18 (27.7%)	
Unsafe	2 (2.0%)	5 (7.7%)	
Environmental factors			
Passenger of other drivers, No. (%)	90 (86.5%)	65 (95.6%)	.067 (1)^b^
Sum of modes of transport used other than the private car, mean (SD)	2.48 (0.83)	2.12 (1.04)	.013 (168)^a^*
Car accidents, mean (SD)	0.10 (0.33)	0.16 (0.51)	.484 (171)^a^
Process of driving cessation			
Driving reduction, mean (SD)	−1.49 (1.49)	− 1.83 (1.72)	.151 (168)^a^
Driving restriction, mean (SD)	1.85 (1.77)	2.34 (2.30)	.343 (170)^a^

^a^Mann-Whitney U test

^b^Fisher’s Exact test

^c^χ2 test

Statistical significance (*p* < .05) is indicated by*

Abbreviations: *AD* Alzheimer’s disease, *CDR-score* Clinical Dementia Rating Total Score, *MMSE-score* Mini Mental State Examination Total Score

Intrapersonal factors that correlated significantly with *StillDriving* were age (*r* = −.156, *p* = .041), gender (*r* = −.153, *p* = .045), CDR-score (*r* = −.437, *p* < .001), MMSE-score (*r* = .309, *p* < .001), health decline (*r* = −.254, *p* = .001), and contrast sensitivity (*r* = .171, *p* = .025). Interpersonal factors that correlated with *StillDriving* included the recommendation given after the fitness-to-drive assessment (*r* = .657, *p* < .001) and recommendations of driving cessation from authority figures (*r* = −.309, *p* < .001). One environmental factor correlated with *StillDriving*, i.e. the sum of modes of transport used other than the private car (*r* = .188, *p* = .015) with retired drivers using less modes of transport than current drivers. Subsequently, the factors correlating significantly with *StillDriving* were entered in a binary logistic regression analysis to determine the validity of the factors in predicting *StillDriving*. A significant model emerged to predict *StillDriving*, χ^2^(9, *N* = 167) = 104.8, *p* < .001. The model explained 46.6% of the total variance (Cox & Snell R^2^) and classified 85.6% of the patients correctly as still driving or not. The factors that contributed significantly to the prediction were gender, CDR-score, health decline, and the recommendation given after the fitness-to-drive assessment, and there was a trend found for recommendations of driving cessation from authority figures (Table 3).

**Table 3:** Summary of binary logistic regression analysis for the prediction of driving continuation (*n* = 101) versus driving cessation (*n* = 66) in patients with cognitive impairment

Predictor variable	B	*SE* B	Wald	P	Odds ratio
Age	0.002	0.002	0.800	.371	1.002
Gender	−1.149	0.575	3.991	.046*	0.317
CDR-score	−4.512	1.498	9.075	.003*	0.011
MMSE-score	−0.026	0.070	.137	.712	0.975
Health decline	−0.658	0.288	5.211	.022*	0.518
Contrast sensitivity	0.201	0.340	.348	.555	1.222
Authority figure recommended driving cessation	−2.149	1.249	2.961	.085	0.117
Recommendation after fitness-to-drive assessment	1.748	0.321	29.724	<.001*	5.743
Sum of other used modes of transport	−0.234	.290	.649	.420	0.792
Constant	−1.101	5.568	.039	.843	0.333
Total R^2^ = 0.466*					

Statistical significance (*p* < .05) is indicated by *

### Driving cessation rates per aetiology of cognitive impairment

At the time of follow-up, 104 (60.5%) patients with cognitive impairment were still driving whereas 68 (39.5%) patients with cognitive impairment had ceased driving. The lowest rate of driving cessation was found in patients with DLB (1 of 8 patients; 12.5%). In patients with PD, the rate of driving cessation was similar (3 of 17 patients; 17.6%). Driving cessation rates were 30.8% (4 of 13 patients) in patients with FTD and 38.2% (32 of 83 patients) in patients with AD. The driving cessation rates were higher in patients with VaD (10 of 15 patients; 66.7%) and AD plus VaD (8 of 10 patients; 80.0%). Of the patients with other or unclear diagnoses, 38.5% ceased driving (10 of 26 patients).

### Mobility of patients with cognitive impairment

#### Important modes of transportation

**Fig. 2:**
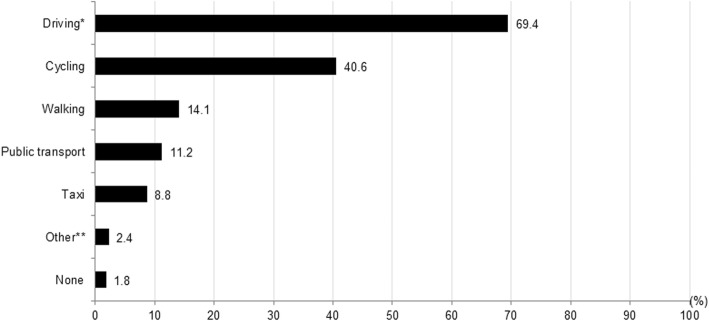
Percentages of patients indicating the importance to continue to use certain modes of transportation (multiple answers possible, *n* = 170). *Driving included both being a driver and being a passenger of a private car. **Other included motorised quadricycles, a motorcycle, and a transportation service of day care

Patients with cognitive impairment (*n* = 170) reported none until up to six modes of transport they found important to continue to use. Driving (i.e. driving themselves or being passenger of other drivers) was by far the most important mode of transportation followed by cycling (Fig. 2).

#### Used modes of transportation

Of the current drivers with cognitive impairment, 86.5% reported also being passenger of other drivers: their partners (63.5%), other family members (28.8%), friends (22.1%), and other drivers such as neighbours or colleagues (3.8%). They also used other modes of transport, especially walking and cycling (Table 4). Of the retired drivers with cognitive impairment, 95.6% reported being passenger of other drivers: their partners (58.8%), other family members (47.1%), friends (17.6%), and other drivers such as a former colleague or a professional caretaker (4.4%). In comparison to current drivers, a smaller proportion of retired drivers was walking, cycling and using public transport and a larger proportion of retired drivers used taxis (Table 4).

**Table 4:** Modes of transportation used by current and retired drivers with cognitive impairment (multiple answers possible)

Mode of transportation	Current drivers (*n* = 104)	Retired drivers (*n* = 68)
Passenger of other driver(s)	86.5%	95.6%
Walking	91.1%	79.4%
Cycling	84.5%	58.8%
Public transport	52.0%	35.3%
Taxis	10.7%	25.0%
Other modes^a^	11.7%	13.2%

^a^Other modes included an airplane, a boat, moped, motorcycle, motorised quadricycle, mobility scooter, buggy at a golf court, and transportation service of day care

##### Changes in frequencies of walking, cycling, and public transport use

**Fig. 3:**
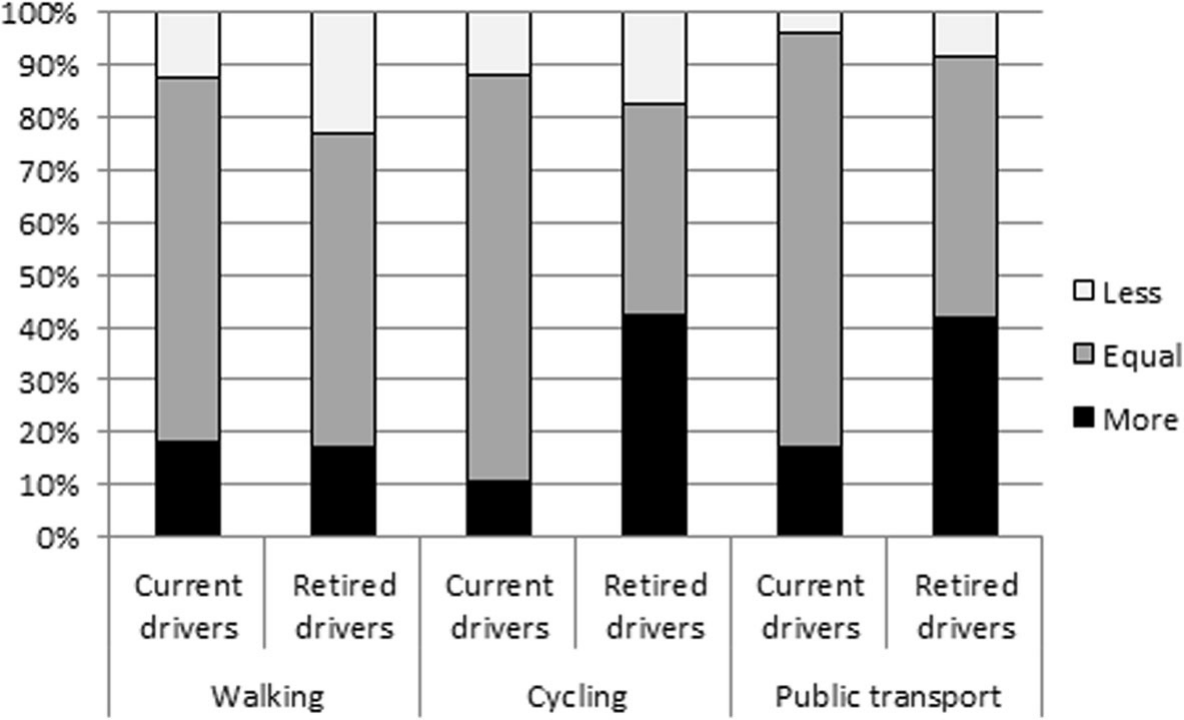
Percentages of current and retired drivers with cognitive impairment who increased, did not change, or decreased their frequency of walking (*n* = 134), cycling (*n* = 125), and use of public transport (*n* = 77) after a fitness-to-drive assessment

The percentages of retired drivers cycling (58.8%) and using public transport (35.3%) were low compared with current drivers (84.5% respectively 52.0%), however, the percentage of retired drivers who increased the frequency of cycling (42.5%) and public transport use (41.7%) after the fitness-to-drive assessment was higher compared with current drivers (10.6% respectively 17.0%) (Fig. 3). These retired drivers mentioned using these modes of transport instead of the car. Nevertheless, the majority did not increase or even decreased the frequency of walking, cycling, and public transport use.

##### Reasons for not walking, cycling, and using public transport

**Fig. 4:**
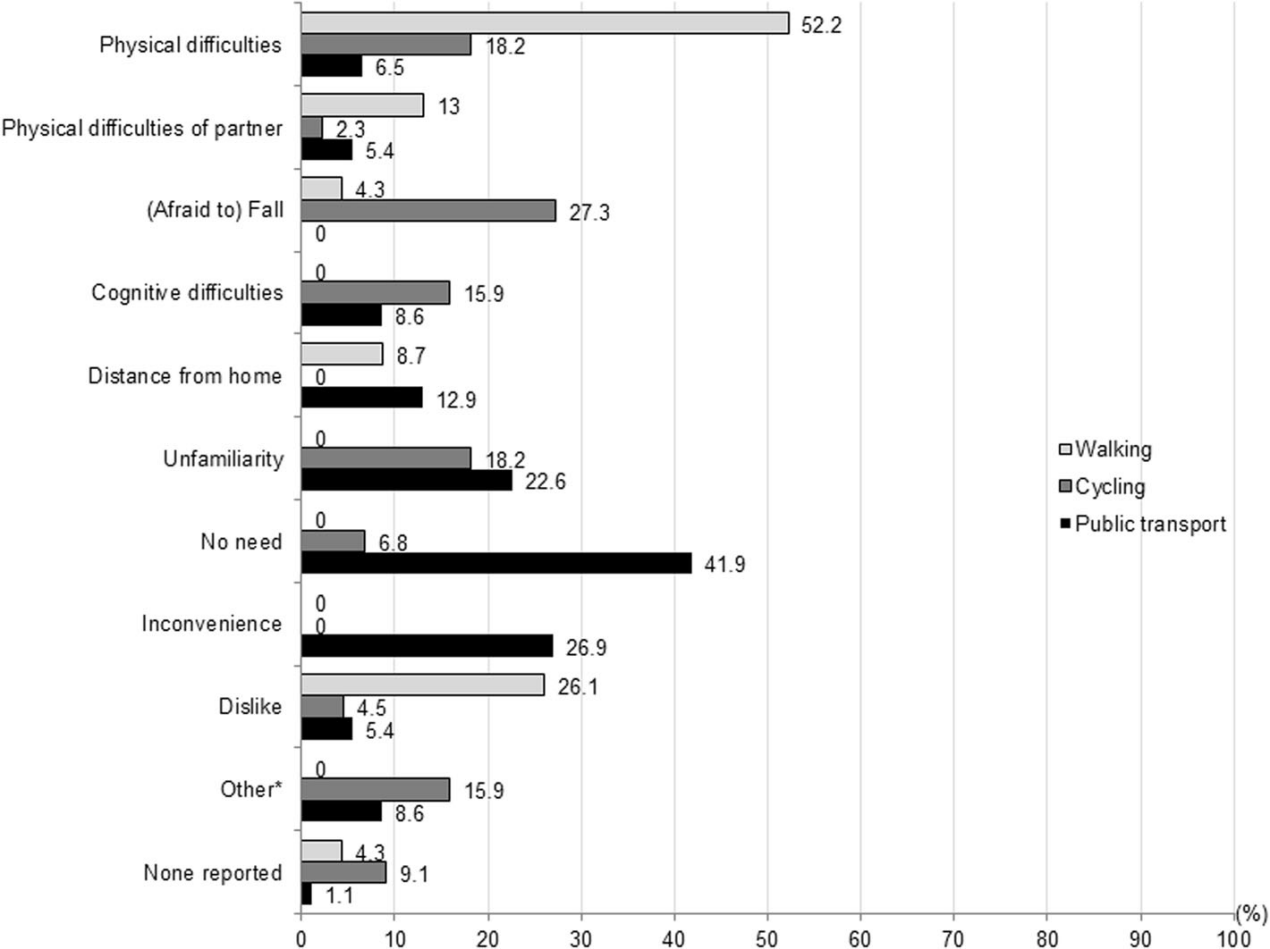
Percentages of reported reasons for not walking (*n* = 23), not cycling (*n* = 44), and not using public transport (*n* = 93) (multiple answers possible). *Other included for cycling: feeling insecure on a bicycle, bicycle got stolen, a cycling accident, being hospitalized, and passiveness, and for public transport: costs, being hospitalized, partner dislikes public transport, experience with severe delay, feels nauseous in public transport, cannot take mobility scooter along, and maintaining driving skills

Patients reported each none up to three reasons for not walking (*n* = 23), not cycling (*n* = 44), and/or not using public transport (*n* = 93) (Fig. 4). Not walking and not cycling was mostly associated with physical difficulties and falls. Dislike was another major reason for not walking for transport, whereas unfamiliarity and cognitive difficulties were other limiting factors for cycling. Not using public transport was largely explained by having no need to use public transport, because of using other modes of transportation. It is noteworthy that inconvenience of public transport was often reported, which could be related to physical difficulties, but also to cognitive difficulties (e.g. impairments in orientation) as well as unfamiliarity and distance from home.

## Discussion

In this study, 172 patients with cognitive impairment were interviewed about their adherence to a driving recommendation received after participation in a comprehensive fitness-to-drive assessment. The vast majority of patients adhered to a recommendation to either continue driving, to follow driving lessons and undergo an official relicensing procedure, or to cease driving after the fitness-to-drive assessment. This indicates that fitness-to-drive assessments promote driving continuation in patients who are fit to drive while stimulating driving cessation in patients who are unfit to drive. Almost 40% of the patients with cognitive impairment ceased driving at follow-up. Nonetheless, some patients were reluctant to cease driving, which concurs with previous studies [46, 47]. In attempt to promote adherence, previously suggested practical strategies were applied in this study, i.e. providing details about the test results and a letter of explanation about how the fitness-to-drive assessment resulted in the driving recommendation, and discussing alternative transportation with those who were recommended to cease driving [27]. Despite the implementation of these strategies, 21% of the patients who were recommended to cease driving did not cease driving, which is a matter of concern..

Driving cessation occurred in most cases in response to a recommendation to cease driving, which was given after the fitness-to-drive assessment, by family members or by authority figures. These results indicate that interpersonal factors are very important for patients with cognitive impairment in the decision making process, which is in correspondence with previous studies in patients with dementia [22, 42]. Hence family members and physicians may have a crucial role in imposing the decision to cease driving on patients who ignore a negative outcome of a fitness-to-drive assessment [20, 48]. Future research should focus on how this can be established effectively without harming the relationship with the patient [24, 49].

Personal factors, i.e. gender, CDR-score, and health decline also play a role in driving cessation. The observed gender effect supports findings from previous studies in which women have been found to cease driving earlier than men [31, 32], but this gender difference was not always found [30]. Future studies should clarify if men are more likely to continue driving when it is no longer safe and if women are more likely to cease driving when it is still safe. Based on the current study, men and women should still be treated equally, because the group of patients who neglected a driving cessation recommendation included both men and women. Cognitive impairment and self-rated health have also been found to predict driving cessation in other studies in which no driving recommendation was given [10, 30, 31, 33]. This implicates that when a decline in health is observed, this should be discussed with the car driver with cognitive impairment. If patients can evaluate their own health decline as incompatible with driving, they might be willing to cease driving. In brief, patients with cognitive impairment who underwent a fitness-to-drive assessment were more likely to cease driving if they were recommended to cease driving, were female, and had relatively severe cognitive impairment and/or pronounced health decline.

Consistent with previous studies [8–10], a considerable proportion of patients with various aetiologies of cognitive impairment continued to drive. Driving cessation was most common among patients with VaD (66.7%) and patients with AD and VaD (80.0%). Contrary to the study of Seiler and colleagues [9] in which patients with DLB had the highest rate of driving cessation (90.9%), in this study patients with DLB had the lowest rate of driving cessation (12.5%). In both studies, the time since diagnosis varied between patients from very recent to several years ago, therefore the patients in this study might have been in a milder stage of DLB than the patients in Seiler and colleagues’ study [9]. An explanation for the discrepancy in findings might be that the severity of cognitive impairment is more important for driving cessation than the aetiology of cognitive impairment. In line with this reasoning, CDR-scores were predictive of driving cessation, which corresponds with previous studies [30, 33]. Nonetheless, patients with different aetiologies of dementia may become unfit to drive due to different driving difficulties resulting from different symptoms [8, 14, 15, 50].

Patients with cognitive impairment preferred to use the private car for transportation, as a driver but also as passenger. This preference was expected because patients were selected on their wish to continue driving, as they are the target group for fitness-to-drive assessments. Especially family members (other than the partner) started to drive retired drivers with cognitive impairment, which is in line with Liddle and colleagues’ argument that driving cessation is a family matter [11]. Remarkably, only a quarter of retired drivers with cognitive impairment used taxis. The group of retired drivers used less alternative modes of transportation than the group of current drivers, which may indicate that cognitive impairment may not only impact on driving but also on feasibility of using alternative transportation. An alternative explanation might be that retired drivers are less healthy in general leading to limitations in mobility. Even though the patient sample as used for the present study was characterized by cognitive impairment, physical difficulties were equally often reported as reason for not cycling or not using public transport, and as the major reason for not walking. On the one hand, retired drivers with cognitive impairment as a group may be frailer than current drivers with cognitive impairment, and the independent mobility of especially retired drivers may be limited and decreasing. On the other hand, around 40% of retired drivers using alternative transportation was able to sustain mobility by increasing their frequency of cycling and public transport use. These patients may represent a physically healthy group within the group of retired drivers with cognitive impairment. Research on traffic safety of patients with cognitive impairments using non-car modes of transportation is lacking, but would be helpful in order to indicate which alternative modes of transport should be advised for patients with cognitive impairment. It is important to note that cyclists and pedestrians are vulnerable road users compared to car drivers, therefore traffic safety of retired drivers with cognitive impairments may be compromised. In conclusion, there is a lot of variation in mobility of patients with cognitive impairment, ranging from having no options for transportation anymore after driving cessation to sustaining mobility through driving or increasing use of alternative modes of transportation.

### Limitations

Driving cessation is a process for many patients with cognitive impairment, but there was only one follow-up moment. Therefore, the eventual consequences of the fitness-to-drive assessment were not fully known yet for all patients, i.e. additional patients might have ceased driving soon after follow-up.

A second limitation is that impairments other than in cognition were not investigated thoroughly. The predictor variable ‘health decline’ is a broad term that includes declines in any aspect related to physical and mental health, however, these aspects were not analysed in more detail. Moreover, patients were screened for minimum visual and motor requirements for driving, but impairments in these domains that are not severe enough to lead to immediate revocation of a driving license could still impact on driving. While two visual variables were included, this study failed to consider variables of motor behaviour. This is problematic as patients with PD, but also DLB and VaD, commonly suffer from motor impairments which could impair driving.

Another limitation concerns the use of the CDR for all patients with cognitive impairment. The CDR was originally developed to determine the severity of AD, and was also shown to be applicable to other aetiologies [51]. Nevertheless, for specific aetiologies of cognitive impairment other cut-offs or specific scales may be more appropriate, such as the Frontotemporal Dementia Rating Scale for FTD [52, 53].

## Conclusions

Severity of cognitive impairment is very relevant for fitness to drive and predictive for driving cessation. Therefore, clinical tools such as the CDR should be used to stage the severity of cognitive impairment in the context of driving recommendations. There is consensus that patients with a CDR-score of 2 or 3 should be recommended to cease driving [12]. Patients with a CDR-score of 1 are less likely to be fit to drive than patients with a CDR-score of 0.5, but for both groups assessments are needed to investigate fitness to drive on an individual basis. Besides patients with more severe cognitive impairment, patients who perceive their health decline, and female patients, are more likely to cease driving, and also recommendations of driving cessation stimulate to do so.

Physicians have a very important role in informing patients about the impact of cognitive impairment on driving, because patients may be compromised in the evaluation of their own functioning and abilities. Physicians should explain that driving cessation will probably become inevitable with the progression of their disease and support patients and their family members in adapting to this change. A proportion of patients will have a wish to continue driving. It is difficult to judge fitness to drive of individual patients in clinical practice [24], therefore referral to fitness-to-drive assessments (e.g. to driving license authorities) is advised. This study showed that adherence to recommendations given after fitness-to-drive assessments is high, thus promoting driving cessation in patients who are unfit to drive while stimulating driving continuation in patients who are fit to drive. Still, physicians should discuss driving and mobility again after a fitness-to-drive assessment to assure that non-adherers are less likely to ignore the given driving recommendation, but also to acknowledge consequences of driving cessation. Depending on the personal situation, patients and their family members may need help in finding alternative modes of transportation to sustain their mobility, or might desire recognition of negative emotions related to driving cessation. Patients with cognitive impairment may benefit from social support groups to ease the process of driving cessation, and from alternative transportation tailored to their needs, e.g. dementia-friendly taxi services.

## Abbreviations

AD: Alzheimer’s disease
CDR: Clinical Dementia Rating
DLB: Dementia with Lewy Bodies
FTD: Frontotemporal dementia
MMSE: Mini-Mental State Examination
Non-AD: Non-Alzheimer’s disease
PD: Parkinson’s disease
VaD: Vascular dementia
